# Investigation into the protective effects of hypaconitine and glycyrrhetinic acid against chronic heart failure of the rats

**DOI:** 10.1186/s12906-022-03632-y

**Published:** 2022-06-16

**Authors:** Liqin Wang, Haiming Deng, Tengyu Wang, Yun Qiao, Jianbing Zhu, Mingfeng Xiong

**Affiliations:** 1 Preventive Treatment Center, The First Affiliated Hospital of Nanchang University, Nanchang, Jiangxi Province China; 2 Institute of Shiceng Pulse, Nanchang University, Nanchang, Jiangxi Province China; 3 Department of Cardiology, The First Affiliated Hospital of Nanchang University, Nanchang, Jiangxi Province China; 4 Jiangxi University of Chinese Medicine, Nanchang, Jiangxi Province China

**Keywords:** Chronic heart failure, Hypaconitine, Glycyrrhetinic acid, Immunohistochemistry, Metabolites

## Abstract

**Background:**

The present study aimed to determine the protective effects of hypaconitine (HA) and glycyrrhetinic acid (GA) against chronic heart failure (CHF) in the rats and to explore the underlying molecular mechanisms.

**Methods:**

The CHF rat model was established by transverse-aortic constriction (TAC) operation. Transthoracic echocardiography and hematoxylin eosin (HE) staining were used to evaluate the pathophysiological and histopathological changes of CHF model. The total cholesterol (TCHO) and triglyceride (TG) levels were determined by ELISA assay. The protein expression of fibroblast growth factor 2 (FGF2), vascular endothelial growth factor A (VEGFA) and endothelial nitric oxide synthase (eNOS) in the rat ventricular tissues was determined by immunohistochemistry. The serum metabolites were determined by LC-MS/MS assay.

**Results:**

After applied the HA + GA, the cardiac tissue and structure were obviously improved, and the HA + GA treatment also significantly reduced the plasma levels of TCHO and TG in the CHF rats. The expression of FGF2 and VEGFA protein was up-regulated and the expression of eNOS protein was down-regulated in the ventricular tissues of CHF rats, which was significantly restored after HA + GA treatment. HA + GA treatment down-regulated serum isonicotinic acid, phosphatidylcholine, cardiolipin, estrogen glucuronide, and glycocholic acid, up-regulated serum sphingosine and deoxycholic acid in the CHF rats.

**Conclusions:**

In conclusion, HA + GA showed protective effects on CHF in the rats, and the HA + GA may exert protective effects by reducing lipid levels, up-regulating the expression of FGF2 and VEGFA proteins, attenuating eNOS protein expression, and modulating metabolic pathways. However, the molecular mechanisms underlying HA + GA-mediated effects still require further examination.

## Background

Chronic heart failure (CHF) is a rapidly growing public health problem that occurs secondary to a common chronic phase of cardiac impairment of multiple etiologies [1]. Atherosclerosis is a major risk factor for CHF and its possible mechanisms include imbalance of lipid metabolism and poor adaptive immune response, leading to chronic inflammation of the arterial vessel wall [2]. When the lipid metabolism is abnormal, it may cause cardiac tissue fibrosis, which is also can be induced by myocardial inflammation. As we all know, cardiac remodeling is an important process during the CHF phase, for it could result in cardiac pressure overload. In all pathological molecular mechanisms, the activation of neurohumoral systems, such as the renin-angiotensin-aldosterone-system (RAAS) is similar with pressure overload [3]. In clinical treatments, the angiotensin-converting enzyme inhibitors, β-adrenergic blockers and diuretics are the main methods for CHF, while long-term use of these chemical drugs may cause adverse effects such as hypotension, fluid depletion and electrolyte depletion [4]. Traditional Chinese medicines (TCMs), such as *Salvia miltiorrhiza* and Ginseng, can promote myocardial contractility, dilate blood vessels, diuresis and inhibit ventricular remodelling [5–7]. Some formulaes have been clinically applied in the treatment of heart failure. The advantage of multi-channel and multi-target of TCM can attenuate adverse effect caused by the genetic defect of western medicine treatment target.

Hypaconitine (HA) is one of the main aconitum alkaloids in TCMs prepared with herbs from the genus *Acotinum.* In TCM, *Acotinum* has the function of warming heart yang for CHF, with both cardiac therapeutic and cardiotoxicity. Hence, in clinic, it is applied together with other herbs to reduce the toxicity, such as *Glycyrrhiza uralensis Fisch, Zingiberis Rhizoma,* et al. It has been found that HA had various biological activities including its effects on cardiovascular system. Yi et al., showed that HA could inhibit calmodulin expression and connexin43 phosphorylation in rat cardiac muscle in vivo [8]. Xie et al., showed that HA could induce QT prolongation via inhibiting KCNH2 potassium channels in conscious dogs [9]. Bai et al., showed that HA could inhibit the apoptosis of endothelial cells via targeting histone deacetylase-high mobility group box-1 pathway [10]. In addition, glycyrrhetinic acid (GA) is one of the main glycyrrhizinic acid in TCMs prepared with herbs from the *Glycyrrhiza uralensis Fisch*. There is a well-known decoction Zhi-Gan-Cao-Tang in TCM, especially for cardiopathy, with *Glycyrrhiza uralensis Fisch* as its primary constitute. That’s to say, *Glycyrrhiza uralensis Fisch* has the functions of ameliorating CHF. In these years, GA has been reported to show cardiac protect actions on reducing myocardial infarction size and improving arrhythmia problems [11]. Besides, GA could exert various biological activities. GA could mitigate prophylaxis induced by mesaconitine in the rats, indicating that pretreatment with GA might reduce the toxicity of mesaconitine in the metabolic level [12]; GA has also been found to be a typical active component of Shaoyao-Gancao Decoction for pain relief [13, 14].

In our previous study, we found that HA and GA, the active components of Fucus and Glycyrrhiza, exerted protective effects against CHF through apoptotic signaling pathways [15]. However, the underlying protective effects of HA and GA in CHF remain unclear.

In this study, we performed ELISA to examine the effects of HA + GA treatment on plasma TCHO and TG levels as well as immunohistochemistry to investigate expression levels of FGF, VEGFA and eNOS protein in CHF rats after HA + GA treatment. In addition, LC-MS/MS analysis used to determine the metabolic differences between the HA + GA group and the Model group and to find potential biomarkers and their possible metabolic pathways associated with CHF.

## Materials and methods

### Animals

30 male specific-pathogen free rats (weight 160–170 g) were purchased from Shanghai Slack Laboratory Animal Co., Ltd., (Shanghai, China). The rats were allowed free access to food and water and were kept on a 12-h light/dark cycle at room temperature (20 ± 2 °C) with constant humidity (50 ± 2%). All animal experimental procedures were approved by the Animal Care and Use Committee of Zhejiang Chinese Medical University and complied with laboratory animal management and use regulations (SYXK (Zhejiang) 2013–184). The study is reported in accordance with ARRIVE guidelines (https://arriveguidelines.org).

### Establishment of CHF model in the rats and treatments

The CHF model was established by transverse-aortic constriction (TAC) method based on previous methods [16]. Briefly, the rats were anesthetized with 2.0% isoflurane (Shenzhen Huabao Medical Supplies Industry & Trade Development Co., Ltd., Shenzhen, China), and then were endotracheally intubated with positive pressure ventilation with a tidal volume of 250 mL at a velocity of 120 breaths per minute by a Small Animal Ventilator (Hallowell, Inc., USA). After opening the chest wall via an upper sternotomy, the aorta was carefully dissected out. A self-regulating “L” needle with the external diameter of approximately 0.9 mm was placed on the aorta, on which a 3–0 prolene ligation was placed between the right and left common carotid arteries. The needle was removed cautiously, and the aortic constriction was then created. After the procedure, the wound was closed, and the animal was allowed to recover from anesthesia. Penicillin (REYOUNG Co. Ltd., Shandong, China) was injected intramuscular once a day for 3 days. The TAC-operated rats were assigned as the model group. The rats in Sham group underwent same procedures with the Model group but without TAC operation [17, 18].

During the first 4 weeks after surgery, rats were allowed free access to food and water. The rats were then randomly divided into three groups: Sham group (*n* = 10), Model group (*n* = 10), HA + GA group (*n* = 10). For our former studies on CHF model were built well, in the transthoracic echocardiography part, we chose randomly 5 rats from each group (*n* = 5). However, in the ELISA and HE staining and immunohistochemistry experiments, in consideration of it may reduce the sample size of Sham group, so we selected 6 samples randomly from the Sham group (*n* = 6), and Model group (*n* = 10), HA + GA group (*n* = 10). Yet, for a better comparison in the untargeted metabolomics analysis, we chose all samples in each group (*n* = 10). According to our previous work [15], the dosage of 2.07 mg/kg HA (Baoji Chenguang Biotechnology Co. Ltd., Xi’an, China) + 25 mg/kg GA (Baoji Chenguang Biotechnology Co. Ltd.) were orally administered to rats of HA + GA group once a day for 1 week, and an equal volume of normal saline was given to the rats from the Sham and Model groups. At one week after drug treatments, transthoracic echocardiography was tested on rats, then the animals were sacrificed and the blood and relevant tissues were collected for further assays.

### Transthoracic echocardiography on CHF rats

An animal heart ultrasound method, as we have done in the previous study [15]. We chose 5 rats randomly from each group to do the transthoracic echocardiography, for the purpose of estimating the LV cardiac remodeling of CHF and observing the effects of HA + GA. We collected the cardiac photographs and data through Vevo2100 imaging system (Visual Sonics Inc., Toronto, Canada).

### Collection of tissue samples

After sacrificing the animals, the blood was collected from the abdominal aortic, and plasma was obtained from supernatant of centrifugation of the blood. The heart tissues were then dissected out and weighed. The left ventricular tissues of the heart were cut into two parts: one part was fixed in 4% paraformaldehyde (PFA) and embedded in paraffin for histological analysis, and the other part was preserved in − 80 °C.

### Enzyme-linked immunosorbent assay (ELISA) for detecting TCHO and TG levels

The TCHO and TG levels in the plasma samples were detected by using ELISA kits for TCHO and TG according to the manufacturer’s instructions.

### HE staining and immunohistochemistry

Heart ventricular tissues were fixed in 4% PFA and embedded in paraffin. Then, sections (3 μm) were sequentially permeabilized with 1% Triton X-100 in phosphate buffered saline (PBS) for 30 min at room temperature, boiled in 100 mM sodium citrate (pH 6.0) three times (6 min each) with 5 min intervals for antigen retrieval, washed in 3% hydrogen peroxide for 30 min to remove endogenous peroxidase. Some of the sections were stained with HE fluid, and some blocked for 1 hour in 5% bovine serum albumin at room temperature. Sections were then incubated with anti-FGF (1:200, # Sc-365, Santa Cruz Biotechnology, Dallas, USA), anti-VEGFA (1:200), and anti-eNOS antibody (1:200) at 37 °C for 60 min at 37∘C. After being washed PBS, the sections were incubated with horseradish peroxidase-conjugated secondary antibodies for 40 min at 37 °C. The tissues were then washed by PBS and incubated in diaminobenzidine substrate (#1901275A, DAKO Co., Ltd., Denmark, Sweden). The sections were finally counterstained with hematoxylin and xylene before being rewashed in ethanol. Images were collected by DAB light microscope (Leica Microsystems Ltd., Wetzlar, Germany). Integrated optical density (IOD) was obtained to represent the relative amount of positive staining, and 5 fields (magnification 100x) were randomly chosen to analyze.

### Untargeted metabolomics analysis

All three groups’ serum samples were collected and tested by the LC-MS system according to the protocol. Chromatographic separations were carried out through an ultra performance liquid chromatography (UPLC) system (SCIEX, UK). In order to check metabolites eluted from the column, the high resolution tandem mass spectrometer TripleTOF5600plus (SCIEX, UK) was applied. And the Q-TOF was detected in both positive and negative ion modes. The online KEGG and HMDB database was chose to explain the metabolites. Then **s**upervised PLS-DA was performed through metaX to differentiate the diverse variables between all groups, and the heat-map analysis of the metabolites was executed by the OmicStudio tools.

### Statistical analysis

All experimental data were presented as the mean ± the standard deviation (SD); one-way analysis of variance (ANOVA) followed by Tukey’s multiple comparison test was performed using SPSS 19.0 statistical software. *P* < 0.05 was considered statistically significant.

## Results

### Effects of HA + GA on CHF rats through transthoracic echocardiography

**Fig. 1:**
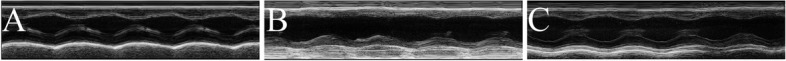
Typical transthoracic echocardiography images from effects of HA + GA on CHF rats. HA: hypaconitine; GA: glycyrrhetinic acid. Sham group (**A**), Model group (**B**), and HA + GA group (**C**)

The results of LV cardiac remodeling in LV end-diastolic dimension (LVIDd), LV end-systolic dimension (LVIDs), were revealled in Fig. 1 and Table 1.

**Table 1:** LV cardiac remodeling levels in Sham, Model and HA + GA group

Group	LVIDd (mm)	LVIDs (mm)
Sham	6.68 ± 0.23	3.63 ± 0.36
Model	8.21 ± 0.19^##^	5.08 ± 0.78^#^
HA + GA	7.33 ± 0.16^**^	3.69 ± 0.67^*^

*HA* hypaconitine, *GA* glycyrrhetinic acid, *LVIDd* LV end-diastolic dimension, *LVIDs* LV end-systolic dimension. Data were presented as mean ± SD (*n* = 5). ^##^*P* < 0.01 versus Sham group; ^∗^*P* < 0.05 and ^**^P < 0.01 versus Model group

Comparing with the Sham group, the values of LVIDd (*P* < 0.01) and LVIDs (*P* < 0.05) were both increased in the Model group. At the same time, when the HA + GA group compared to the Model group, the values of LVIDd (*P* < 0.01) and LVIDs (*P* < 0.05) were both descended. This results indicated that the LV cardiac remodeling of CHF were obviously ameliorated by the HA + GA treatment.

### Observed effects of HA + GA on rat CHF model by HE staining

**Fig. 2:**
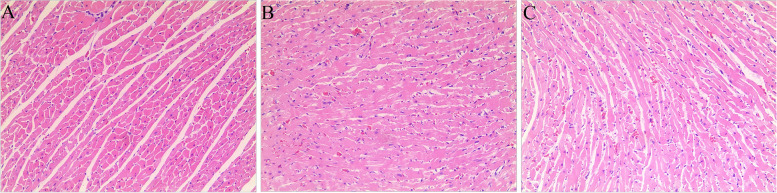
HE staining in the heart tissues of the CHF rats. Sham group(**A**), Model group (**B**), HA + GA group (**C**); (magnification = 100x)

As showed in the Fig. 2, in the Sham group (A), the myocardial tissue structure and cytosolic nucleus were clear. The cardiac muscle fibers were pink and arranged in good order. Meantime, as revealed in the Model group (B), the myocardial tissue structure and cytosolic nucleus were unclear. With the broken and disarranged cardiac muscle fibers, and inflammatory cell infiltration. However, after the treatment of HA + GA (C), compared with Model group, the myocardial tissue structure and cytosolic nucleus were much more clear, and the cardiac muscle fibers were arranged more regularly.

### Analysis of lipid profile in the rat plasma

The effects of HA + GA on TG and TCHO levels in CHF rats were determined by ELISA assay. As shown in Table 2, the TCHO and TG levels were significantly lower in the HA + GA group than that in the Model group. In addition, TG and TCHO levels were significantly lower in the Sham group than that in the Model group (Table 2). The difference in TCHO and TG levels between the HA + GA group and the Sham group was not statistically significant (Table 2).

**Table 2:** TCHO and TG levels in Sham, Model and HA + GA group

Group	TCHO (mg/dl)	TG (mmol/L)
Sham	1.44 ± 0.13	0.31 ± 0.11
Model	2.06 ± 0.29^##^	0.69 ± 0.26^##^
HA + GA	1.68 ± 0.16^**^	0.39 ± 0.07^*^

*HA* hypaconitine, *GA* glycyrrhetinic acid. Data were presented as mean ± SD, Sham group (*n* = 6), Model group (*n* = 10), and HA + GA group (*n* = 10). ^##^*P* < 0.01 versus Sham group; ^∗^*P* < 0.05 and ^**^P < 0.01 versus Model group

### Immunostaining analysis of FGF2, VEGFA and eNOS in the heart tissues of the rats

**Fig. 3:**
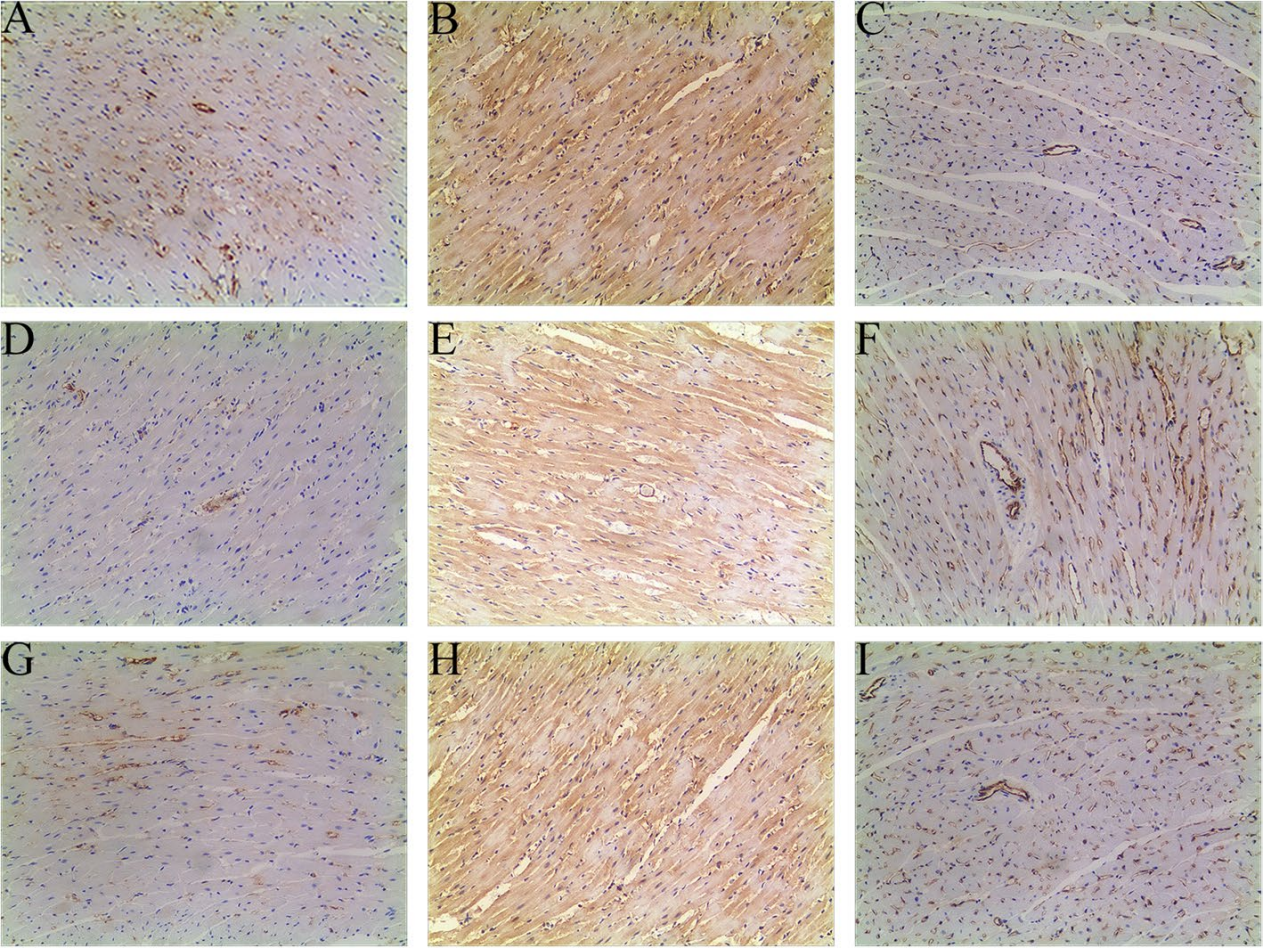
Immunostaining analysis of FGF2, VEGFA and eNOS in the heart tissues of the rats. **A-C** Immunohistochemical staining micrographs of FGF2 (**A**), VEGFA (**B**) and eNOS (**C**) in the rat heart tissues from Sham group (magnification = 100x). **D-F** Immunohistochemical staining micrographs of FGF2 (**D**), VEGFA (**E**) and eNOS (**F**) in the rat heart tissues from Model group (magnification = 100x). **G-I D-F** Immunohistochemical staining micrographs of FGF2 (**D**), VEGFA (**E**) and eNOS (**F**) in the rat heart tissues from HA + GA group (magnification = 100x)

**Fig. 4:**
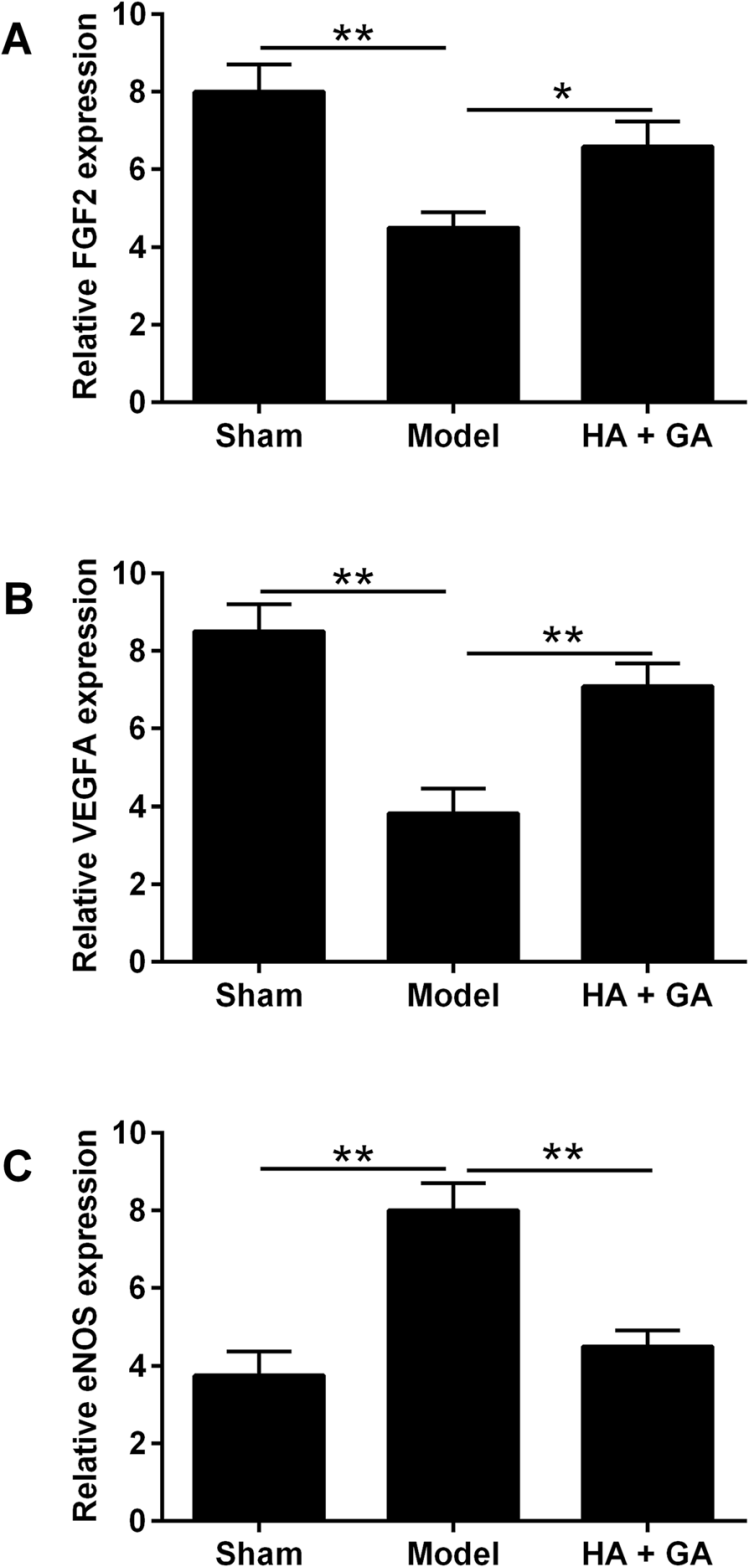
Quantitative analysis of FGF2, VEGFA and eNOS protein expression levels in the rat heart tissues. **A** Quantitative image analyses of FGF2 protein were performed based on the IOD of positive immunostaining (brown) in the heart tissues from Sham, Model and HA + GA group. **B** Quantitative image analyses of VEGFA protein were performed based on the IOD of positive immunostaining (brown) in the heart tissues from Sham, Model and HA + GA group. **C** Quantitative image analyses of eNOS protein were performed based on the IOD of positive immunostaining (brown) in the heart tissues from Sham, Model and HA + GA group. Data are presented as mean ± SD, Sham group (*n* = 6), Model group (*n* = 10), and HA + GA group (*n* = 10). Significant differences between treatment groups were shown as ^*^P < 0.05 and ^**^P < 0.01

Immunohistochemistry was used to detect the effect of HA + GA on the expression of FGF2, VEGFA and eNOS proteins in heart tissues of rats with CHF. As shown in Figs. 3 and 4, the protein expression levels of FGF2 and VEGFA were significantly higher and the protein expression level of eNOS was significantly lower in the HA + GA group than that in the Model group. In addition, the protein expression levels of FGF2 and VEGFA were significantly higher and the protein expression level of eNOS was significantly lower in sham group than that in the model group. There was no statistically significant difference in FGF2, VEGFA and eNOS between the Sham and HA + GA group. The above results indicated that the protective effect of HA + GA in rats with CHF was related to the regulation of FGF2, VEGFA and eNOS protein expression.

### Untargeted metabolomics analysis results

**Fig. 5:**
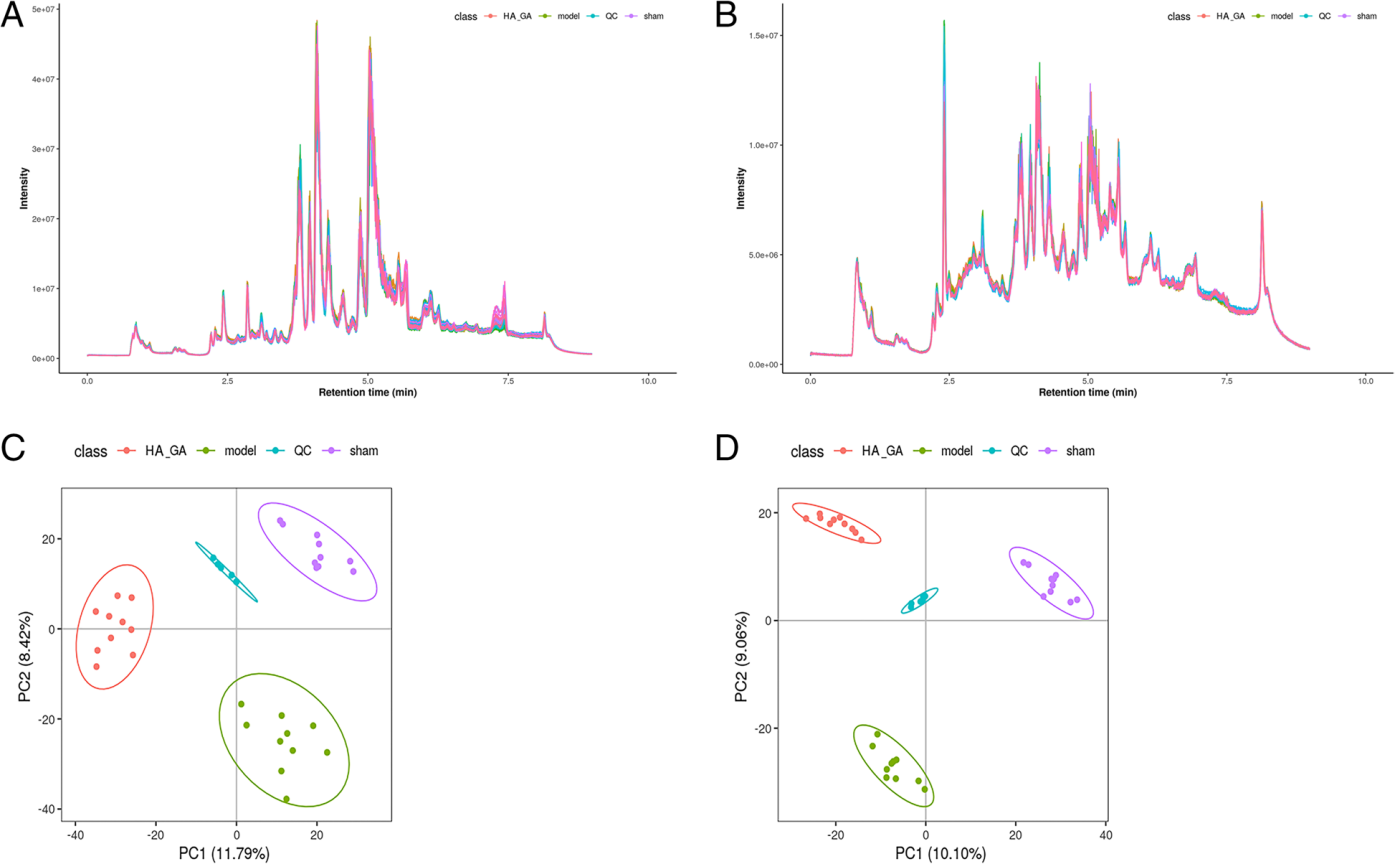
LC/MS-MS analysis of serum metabolites of rats. (**A** and **B**) Total Ion Chromatography) plots in positive (**A**) and negative (**B**) ion modes. (**C-D**) PCA plots in positive (**C**) and negative (**D**) ion modes

The serum metabolites of the rats with different treatments were analyzed by using LC-MS/MS technique. Based on the LC-MS/MS analysis, we detected 9237 metabolite signatures of 9920 metabolites in positive and negative mode (Fig. 5). Figure 5A and B illustrated total ion flow chromatogram (TIC) in positive and negative mode, respectively, and the response intensity and retention time of peaks of each sample largely overlapped. Using unsupervised principal component analysis, QC samples were used to assess the quality of the experiment. as shown in the Fig. 5C and D, the distribution points of QC samples were clustered, indicating good reproducibility of the instrument.

**Fig. 6:**
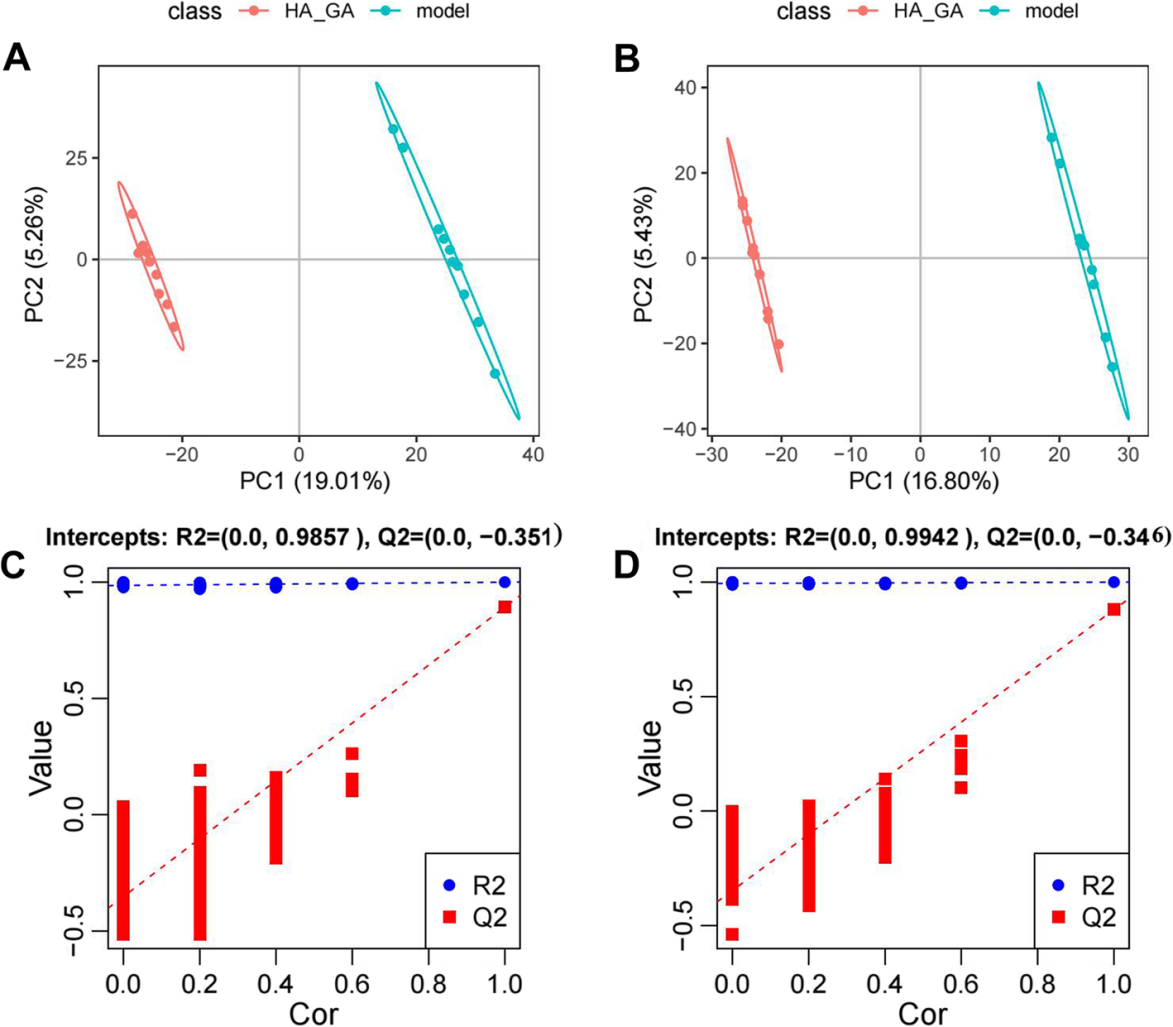
PLS-DA analysis of serum metabolites from Model and HA + GA group. **A** and **B** (PLS-DA) plots of HA + GA group and Model group in positive (**A**) and negative (**B**) ion modes. **C** and **D** The corresponding substitution test plots in the positive (**C**) and negative (**D**) ion models

To better distinguish the differences between groups, we performed the supervised PLS-DA, which enables the prediction of sample categories while reduces the dimensionality. Figure 6A and B demonstrated the PLS-DA results for the comparison between the Model group and HA + GA group. In the results, each point represents a sample, and the clustered sample points of the HA + GA group are remarkably distinct from that of the model group, indicating that there is a significant difference between the Model and HA + GA group. The replacement test of the PLS-DA also showed that the PLS-DA was not overfitted in our datasets (Fig. 6C and D).

**Fig. 7:**
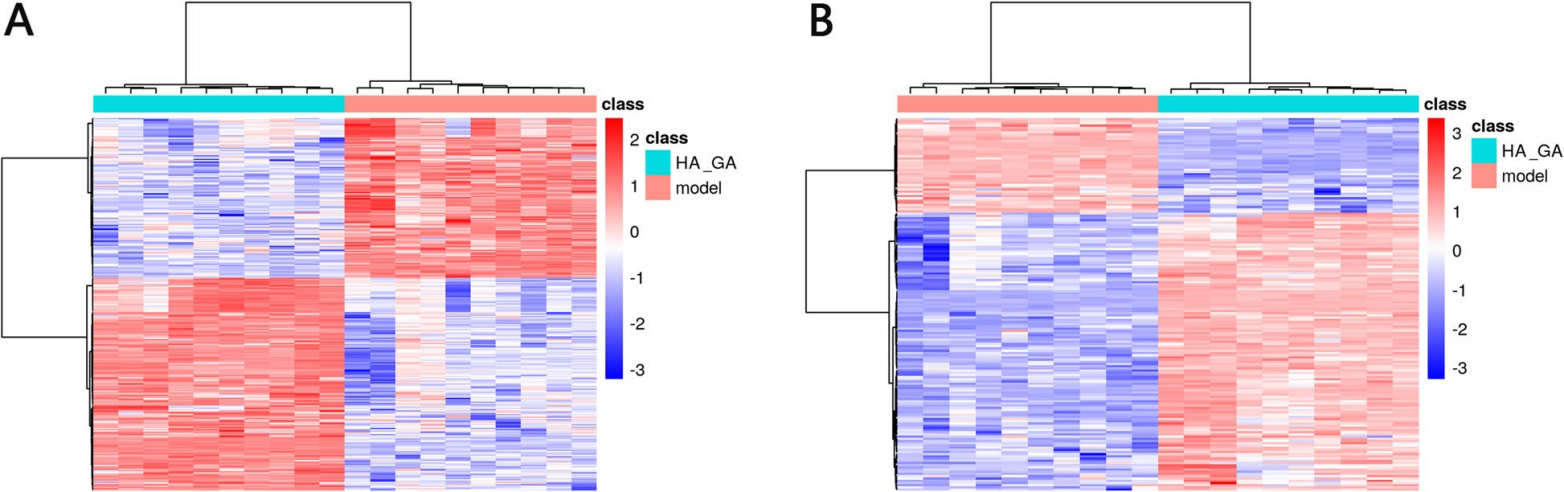
Heatmap plots of the differential serum metabolites between Model and HA + GA group (**A** and **B**) Heatmap plots of differential serum metabolites between HA + GA group and Model group in positive (**A**) ion mode and negative (**B**) ion mode, respectively

Furthermore, hierarchical cluster analysis of the differential metabolites was performed to examine the metabolite subgroups, and the results were presented as a heatmap. Figure 7 demonstrated the heatmap of the hierarchical cluster analysis in positive ion mode and negative ion mode between the HA + GA group and the model group, and highly expressed metabolites were found in both the Model group and HA + GA group. Based on the PLS-DA analysis, a total of 303 differential metabolites in the positive ion mode and a total of 170 differential metabolites in the negative ion mode between Model and HA + GA group were identified.

Furthermore, the associated metabolic pathways of the differential metabolites between Model and HA + GA group were also explored. As shown in the Table 3, isonicotinic acid, phosphatidylcholine, cardiolipin, estrogen glucuronide, and glycocholic acid were significantly up-regulated, while sphingosine and deoxycholic acid were significantly down-regulated in the HA + GA group compared with the Model group. The associated metabolic pathways included sphingolipid metabolism, sphingolipid signaling pathway, and glycerophospholipid metabolism (Table 3).

**Table 3:** Differential metabolites and their metabolic pathways between the Model and HA + GA group

MS2kegg	RT	M/Z	Formula	Regulated	Identification	Pathway
C07446	1.07	124.0387	C6H5NO2	up	Isonicotinic acid	Drug metabolism - other enzymes
C00319	3.20	300.2893	C18H37NO2	down	sphingosine	Sphingolipid metabolism
C00319	3.20	300.2893	C18H37NO2	down	sphingosine	Metabolic pathways
C00319	3.20	300.2893	C18H37NO2	down	sphingosine	Sphingolipid signaling pathway
C00319	3.20	300.2893	C18H37NO2	down	sphingosine	Apoptosis
C00157	4.96	580.3945	C10H18N08PR2	up	Phosphatidylcholine	Glycerophospholipid metabolism
C00157	4.96	580.3945	C10H18N08PR2	up	Phosphatidylcholine	Arachidonic acid metabolism
C00157	4.96	580.3945	C10H18N08PR2	up	Phosphatidylcholine	Linoleic acid metabolism
C00157	4.96	580.3945	C10H18N08PR2	up	Phosphatidylcholine	alpha-Linolenic acid metabolism
C00157	4.96	580.3945	C10H18N08PR2	up	Phosphatidylcholine	Metabolic pathways
C00157	4.96	580.3945	C10H18N08PR2	up	Phosphatidylcholine	Retrograde endocannabinoid signaling
C00157	4.96	580.3945	C10H18N08PR2	up	Phosphatidylcholine	Choline metabolism in cancer
C04483	3.33	391.2854	C24H40O4	down	Deoxycholic acid	Bile secretion
C11133	2.35	445.1907	C24H30O8	up	Estrone glucuronide	Steroid hormone biosynthesis
C01921	2.83	464.3029	C26H43NO6	up	Glycocholic acid	Primary bile acid biosynthesis
C01921	2.83	464.3029	C26H43NO6	up	Glycocholic acid	Metabolic pathways
C01921	2.83	464.3029	C26H43NO6	up	Glycocholic acid	Bile secretion
C05980	5.13	483.2734	C13H18O17P2R2	up	Cardiolipin	Glycerophospholipid metabolism

## Discussion

We aimed to investigate the protective effects of the active ingredients of Aconite and *Glycyrrhiza glabra* on rats with CHF. Firstly, we chose transthoracic echocardiography and HE staining to estimate effects of HA + GA on CHF rats. Then, we performed ELISA to examine the effects of HA + GA treatment on plasma TCHO and TG levels as well as immunohistochemistry to investigate expression levels of FGF, VEGFA and eNOS protein in CHF rats after HA + GA treatment. In addition, LC-MS/MS analysis used to determine the metabolic differences between the HA + GA group and the Model group and to find potential biomarkers and their possible metabolic pathways associated with CHF.

The biochemical tests revealed that TCHO and TG levels in Model were higher than in the HA + GA group, while the difference between the HA + GA and Sham groups was not statistically significant, suggesting that the protective effect of HA + GA on chronic heart failure was associated with a reduction in lipid levels. In hypercholesterolemic patients, plasma FGF2 levels were significantly lower, which was associated with the inhibitory effect of plasma low-density lipoprotein (LDL) [19]. In atherosclerotic lesions, VEGFA expression expressed in smooth muscle cells was significantly increased [20]. It is well known that eNOS has atherosclerotic effects through the synthesis of NO, which has been shown to promote atherosclerosis by oxidizing LDL. The immunohistochemistry results showed that the HA + GA group achieved a protective effect against CHF by up-regulating the expression of FGF2 and VEGFA proteins and attenuating eNOS protein expression.

Sphingolipids are a complex class of lipids consisting of a backbone sphinx base that can be phosphorylated, acylated, glycosylated, bridged to various head groups via phosphodiester bonds, or otherwise modified, resulting in hundreds of sphingolipid subspecies [12]. Sphingolipids have been found to be closely associated with cardiovascular disease; and sphingomyelin levels are significantly increased in atherosclerotic lesions [13], and Poss AM et al., showed that its serum levels have been suggested as a biomarker for cardiovascular disease [14]. Sphingosine is a biologically active signaling molecule among sphingolipid metabolites, and we observed that sphingosine levels were significantly downregulated in the HA + GA group compared to that in the Model group, and we speculated that HA + GA may achieve protective effects in rats with CHF by modulating sphingolipid metabolic pathways and sphingolipid signaling pathways.

Phosphatidylcholine is a common glycerophospholipid that is widely found in animal and plant tissues and egg yolk. The choline component of dietary phosphatidylcholine is metabolized by human intestinal microorganisms to produce trimethylamine-N-oxide (TMAO), which increased the risk of cardiovascular disease [21]. Paapstel K et al. found a negative correlation between serum phosphatidylcholine levels and vascular injury in patients with atherosclerosis [22]. We found that phosphatidylcholine levels were significantly upregulated in the HA + GA group compared to that in the Model group, suggesting that HA + GA achieves vascular protection in CHF rats by promoting the synthesis of phosphatidylcholine. Cardiolipin is a phospholipid specific to mitochondrial membranes and plays an important role in the composition and function of mitochondrial membranes. Impaired energy metabolism is a feature of heart failure, which is associated with iron deficiency and impaired mitochondrial function in patients with heart failure [16]. The important function of cardiolipin for mitochondria lies in its combined action on protein complexes and super complexes [23]. Even for green plants, cardiolipin plays an important role in the mitochondrial development and function [24]. We found that cardiolipin levels were significantly up-regulated in the HA + GA group, indicating that HA + GA achieved a protective effect against CHF in rats by modulating the glycerophospholipid pathway. In addition to regulating sphingolipid metabolic pathway and glycerophospholipid metabolic pathway, it is also related to regulate sphingolipid signaling pathway, apoptosis, arachidonic acid metabolism, linoleic acid metabolism, α-linolenic acid metabolism, and bile secretion pathway.

## Conclusions

In conclusion, HA + GA showed protective effects on CHF in the rats, and the HA + GA may exert protective effects by reducing lipid levels, up-regulating the expression of FGF2 and VEGFA proteins, attenuating eNOS protein expression, and modulating metabolic pathways. However, the molecular mechanisms underlying HA + GA-mediated effects still require further examination. This paper also can give abundant theoretical basis for the clinical application of *Acotinum* combined *Glycyrrhiza uralensis Fisch.* Moreover, our results could supply new viewpoints for treatment targets on CHF.

## Data Availability

All the data generated and analyzed in this study are mentioned in this manuscript.

## Abbreviations

ANOVA: One-way analysis of variance
CHF: Chronic heart failure
ELISA: Enzyme-linked immunosorbent assay
eNOS: Endothelial nitric oxide synthase
HA: Hypaconitine
FGF2: Fibroblast growth factor 2
IOD: Integrated optical density
LDL: Low-density lipoprotein
LVIDd: LV end-diastolic dimension
LVIDs: LV end-systolic dimension
GA: Glycyrrhetinic acid
PFA: Paraformaldehyde
RAAS: Renin-angiotensin-aldosterone-system
SD: Standard deviation
TAC: Transverse-aortic constriction
TCHO: Total cholesterol
TCMs: Traditional Chinese medicines
TG: Triglyceride
TIC: Total ion flow chromatogram
UPLC: Ultra performance liquid chromatography
VEGFA: Vascular endothelial growth factor A
